# Electricity generation from paddy soil for powering an electronic timer and an analysis of active exoelectrogenic bacteria

**DOI:** 10.1186/s13568-019-0781-x

**Published:** 2019-04-23

**Authors:** Yu Lu, Li Liu, Shaosong Wu, Wenhui Zhong, Yujun Xu, Huan Deng

**Affiliations:** 1 School of Environment, Nanjing Normal University, Nanjing, 210023 China; 2 Jiangsu Provincial Key Laboratory of Materials Cycling and Pollution Control, School of Geography Sciences, Nanjing Normal University, Nanjing, 210023 China; 3 Jiangsu Center for Collaborative Innovation in Geographical Information Resource Development and Application, Nanjing, 210023 China; 4 Honors College, Nanjing Normal University, Nanjing, 210023 China

**Keywords:** Paddy soil, Microbial fuel cells, Exoelectrogenic bacteria, Electricity, Power

## Abstract

In farmlands, most electronic devices have no connection to a power source and have to work on batteries. To explore paddy soil as an in situ power source, herein, we in the present study constructed sediment microbial fuel cells (SMFCs) in paddy soil. An open circuit voltage of 1.596 V and a maximum power density of 29.42 mWm^−2^ were obtained by serially connecting three SMFCs. Electrochemical impedance spectroscopy showed that the internal resistance which comprised ohmic resistance and anodic and cathodic charge transfer resistance was approximately 400 Ω for each of the three individual SMFCs. We used the serially connected SMFCs to power an electronic timer through a 1 F capacitor. The SMFCs had powered the timer for 80 h until the potential of the SMFCs dropped below 0.936 V. Then, RNA was extracted from anode samples and 16S rRNA was sequenced following reverse transcription. The results showed that the relative abundance of active exoelectrogenic bacteria-associated genera on the anode was 13.03%, 27.78%, and 16.17% for the three SMFCs with *Geobacter* and *Anaeromyxobacter* being the dominant genera. Our findings provide the possibility of powering electronic devices in the field by using soil as a power source.

**Electronic supplementary material:**

The online version of this article (10.1186/s13568-019-0781-x) contains supplementary material, which is available to authorized users.

## Introduction

It is known that rice, corn and wheat are the top three crops in the world. To ensure the quantity and quality of rice production, an increasing number of paddy fields are monitored by using a series of electronic devices for soil pollution (Biyani et al. 2017), soil water dynamics (Chiaradia et al. 2015), and growth status (Bai et al. 2018). In addition, paddy fields have been monitored more often than before for greenhouse gases emissions (Zhou et al. 2016). However, a power source is usually unavailable in farmland, especially in remote areas. Although a battery is easy to carry, it provides limited electricity. One possible solution is to explore applicable paddy soil based sediment microbial fuel cells (SMFCs) to provide in situ and auxiliary electrical power.

Paddy soil has diverse and abundant exoelectrogenic bacteria because of its anoxic environment with organic matters as electron donors and Fe(III)/Mn(IV) as electron acceptors (Yuan et al. 2018). A number of genera that contain exoelectrogenic bacteria species have been detected in paddy soils, such as *Geobacter*, *Clostridium*, *Comamonas*, *Anaeromyxobacter*, and *Bacillus*. These exoelectrogenic bacteria-associated genera account for 0.5–7.7% of the total bacteria in abundance (Wang et al. 2019). There have been several attempts to demonstrate that paddy soil can generate electricity in microbial fuel cells (MFCs). The most-used MFCs configuration is SMFCs, in which the anode is embedded in the paddy soil and the cathode is located in the overlaying water, using dissolved oxygen as an electron acceptor. Owing to limited organic substrates, low conductivity and high internal resistance, paddy soil-based SMFCs generally produce an open circuit voltage (OCV) of approximately 500 mV and a maximum power density of less than 10 mWm^−2^ (Deng et al. 2014; Wang et al. 2015). Several efforts have been made to improve the performance of soil-based SMFCs, including growing rice plants in SMFCs (Kaku et al. 2008; Deng et al. 2012; Kouzuma et al. 2014), amending paddy soil with organic substrates (Takahashi et al. 2016), and increasing the NaCl concentrations (Miyahara et al. 2015). These efforts resulted in an elevated OCV of 800 mV, which seems to be the limit for paddy soil-based SMFCs and is still too low to power electronic devices.

In the present study, we constructed serially connected paddy soil-based SMFCs to obtain an elevated OCV and attempted to use the SMFCs to run an electronic timer. Serially connected SMFCs sometimes meet a problem of voltage reversal, a phenomenon that the voltage of an individual MFC in a serially-connected MFCs reversed from a positive to a negative value (An and Lee 2014). Substrates starvation is considered to be the main cause for voltage reversal and adding substrates is a promising way to prevent voltage reversal (Oh and Logan 2007). Therefore, to enhance the production of electrons, maintain the negative anode potential and avoid the occurrence of voltage reversal, we in this study added straw powder into the paddy soil to provide sufficient fuel for the exoelectrogenic bacteria. In addition, porous carbon felt, which can provide abundant reaction sites for exoelectrogenic bacteria, was used as the anode, and platinum, which has the highest catalysis efficiency for the reduction of oxygen, was used as the cathode (Mateo et al. 2017). Despite knowing the abundance and diversity of the exoelectrogenic bacteria-associated genera in paddy soil (Wang et al. 2019), it is not clear which genera are active and might contribute the most to power generation. Thus, we extracted RNA from anode samples, followed by high-throughput sequencing. The aims of the present study were to (1) explore paddy soil as a novel energy source to power an electronic device, and (2) identify the active exoelectrogenic bacteria-associated genera on the anode.

## Materials and methods

### Soil sampling and physiochemical properties

Soil (0–20 cm in depth) was sampled in July 2018 from a flooded paddy field in Nanjing city (N32°05′18″; E118°59′28″). After sampling, the soil was sieved and passed through a 2 mm mesh. The sieved fresh soil was stored at 4 °C for the following “Construction and operation of SMFCs” experiment. Part of the sieved soil was air-dried for physiochemical analysis according to Wu et al. (2018). The soil physiochemical properties are as follows: soil pH, 7.26; soil electrical conductivity, 105.41 μS cm^−1^; soil total carbon, 30.58 g kg^−1^; and soil total nitrogen, 2.98 g kg^−1^.

### Construction and operation of SMFCs

**Fig. 1:**
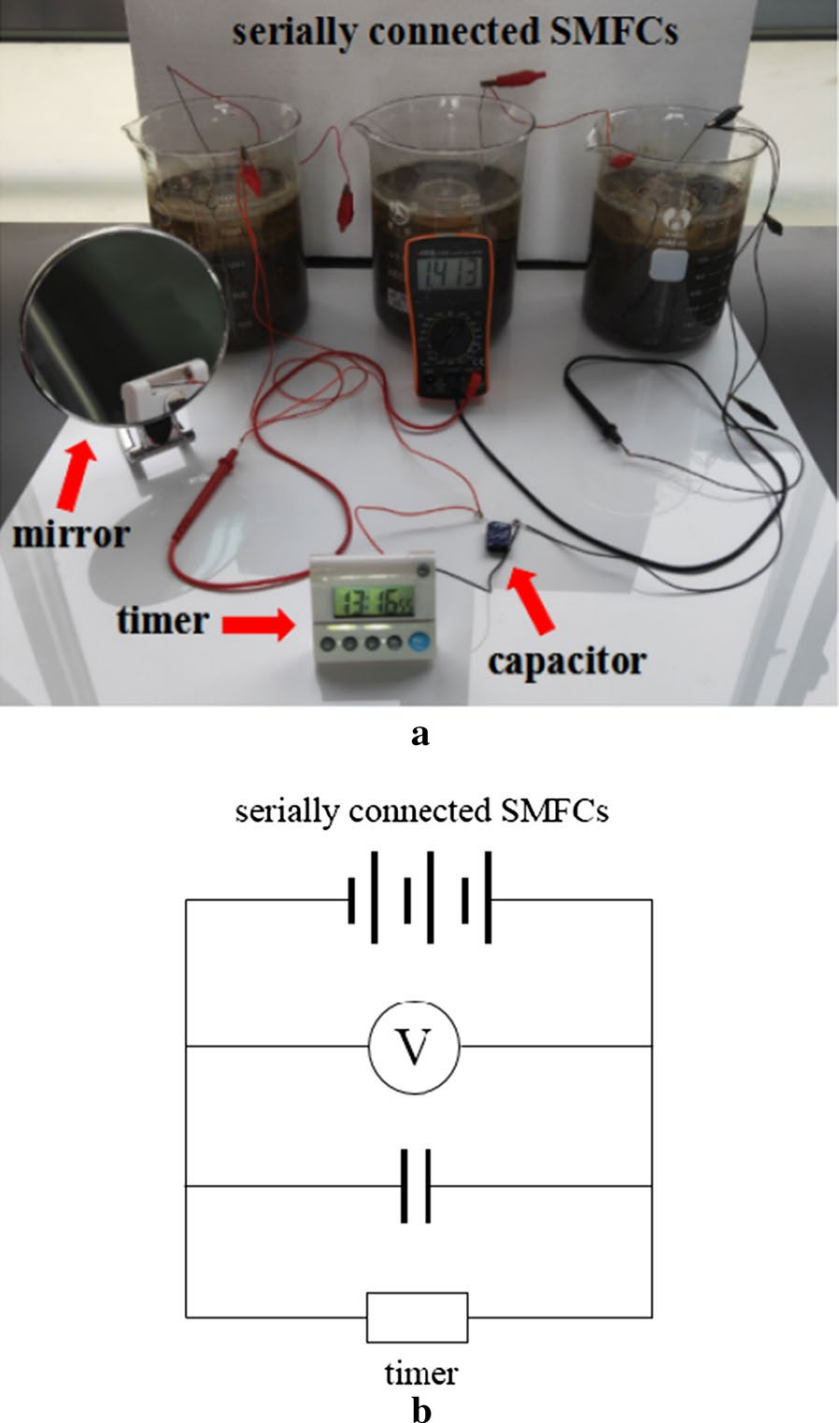
**a** A photo of the serially connected sediment microbial fuel cells (SMFCs) and **b** circuit diagram. The voltmeter shows a voltage of 1.413 V over the timer, which has been running for 13 h 16 min 55 s. The mirror shows the battery box of the timer, indicating that the battery was replaced by the SMFCs

Three SMFCs (denoted as SMFC1, SMFC2 and SMFC3) were constructed in three beakers with dimensions of 14 cm × 19 cm (diameter × height). The anode was carbon felt with diameter of 7 cm and thickness of 0.5 cm, and the cathode was platinum mesh with a diameter of 4.5 cm. Each beaker was packed with 1500 g of soil (dry weight), and the anode was embedded at 5 cm below soil surface. Water was added gently to the soil until the water surface was 3 cm above the soil surface and the cathode was submerged into the overlaying water. The anode and cathode of each SMFC were connected with a 1000 Ω external resistor for acclimation of the exoelectrogenic bacteria, until the voltage curve reached a plateau. Then, the external resistors were removed and the three SMFCs were serially connected. When the open circuit voltage (OCV) of the serially connected SMFCs exceeded 1.5 V, a 1 F capacitor was connected with the SMFCs until it was fully charged. Finally, an electronic timer with a rated voltage of 1.0 V and rated power of 18 μW was connected in parallel to the capacitor and serially connected SMFCs so that it could be powered on and started running (Fig. 1). The SMFCs were operated at room temperature, which was recorded using an electronic thermometer (T12R-EX, HuaHanWei, Shenzhen, China) every 15 min (Additional file 1: Figure S1).

### Electrochemical measurements

The voltage data of each individual SMFC and the serially connected SMFCs were recorded every 15 min using a data acquisition module. After the electronic timer finished running, the polarization and power curves of the individual SMFCs and series-connected SMFCs were obtained by varying external resistances sequentially from 20 k, 10 k, 5 k, 1 k, 500, 100, 50, 10, to 5 Ω at 15-min intervals (Lee et al. 2017). The current density and power density were calculated from *j* = *U*/(*RA*), *P *= *UI*/*A*, where *j* is the current density (mAm^−2^), *P* is the power density (mWm^−2^), *U* (V) is the voltage, *R* is the external resistance (Ω), *I* is the current (A), and *A* (m^2^) is the projected surface area of the anode.

Electrochemical impedance spectroscopy (EIS) was performed using an electrochemical workstation (Versa STAT4, Princeton applied research, Oak Ridge, US). A two-electrode system was constructed to test the internal resistance of an individual SMFC with the anode serving as a working electrode, and the cathode as a counter electrode (Deng et al. 2014). However, it is difficult to discriminate the charge transfer resistance (*R*_ct_) of the anode from that of the cathode based on the two-electrode system. To resolve this problem, a three-electrode system was constructed to examine cathodic *R*_ct_ with an Ag/AgCl electrode serving as a reference electrode. Therefore, the *R*_ct_ obtained from the two-electrode system, largely different from a cathodic *R*_ct_, is considered to be an anodic *R*_ct_. The EIS was operated over a frequency range of 10 mHz–100 kHz with an amplitude of 10 mV. Nyquist plots were fitted into equivalent circuits using ZsimpWin software.

#### RNA extraction and reverse transcription

After the electrochemical measurements, the anode from each individual SMFC was gently rinsed to remove the soil by using sterilized distilled water. Then, a square piece of anode (side length = 1.5 cm) was collected and subjected to RNA extraction by using the Fast RNA SPIN kit for soil (MP biomedicals, Solon, US). RNA extraction was confirmed as DNA-free by running PCR with a primer set 27F/907R (Chen et al. 2007). The quantity of the extracted RNA was determined using a nanodrop UV–Vis spectrophotometer (ND-1000, NanoDrop Technologies, Wilmington, US). Single-strand complementary DNA (cDNA) was synthesized immediately after the RNA extraction. The 10 μL reaction mixture contains 1 μL of total RNA, 2 μL of 5 × PrimeScript™RT Master Mix, and 7 μL of RNase free distilled water. The mixture was incubated at 37 °C for 15 min for cDNA synthesis, and then at 85 °C for 5 s to inactivate reverse transcriptase. The cDNA were stored at − 80 °C before use.

### High-throughput sequencing

To characterize the composition of the potentially active exoelectrogenic bacteria-associated genera, cDNA samples from the three individual SMFCs were subjected to high-throughput sequencing targeting the V4–V5 regions of the 16S rRNA gene using the universal primer set 515 F and 907 R (Biddle et al. 2008). The amplification of the cDNA was performed in a 25 μL reaction mixture containing 2.5 U of Taq DNA polymerase (Sangon Biotech, Shanghai, China), 1 μL of each primer (10 μM), 2.5 μL of 10X buffer (supplemented with Mg^2+^), 4 μL of dNTPs mixture (2.5 mM of each dNTP), and 1 μL of cDNA as the template. The thermal cycling conditions were as follows: 95 °C for 3 min, followed by 40 cycles of 10 s at 95 °C, 20 s at 55 °C, and 20 s at 72 °C. The amplification products were visualized by electrophoresis. Finally, paired-end high-throughput sequencing was performed on an Illumina MiSeq PE300 platform at Majorbio (Shanghai, China). The sequences data were processed according to Wang et al. (2019) and were deposited in the NCBI sequence read archive (SRA) database (Bio-project number: PRJNA511515; SRA accession: SRP174322).

Because there is still no universal primers for exoelectrogenic bacteria, it is impossible to quantify them by a molecular method. Nevertheless, we reported a method that could provide a close estimation of the abundance of exoelectrogenic bacteria by quantifying exoelectrogenic bacteria-associated genera, which was defined as the genera that contain exoelectrogenic bacterial species (Wang et al. 2019). In this study, the relative abundance of active exoelectrogenic bacteria was estimated based on the relative abundance of exoelectrogenic bacteria-associated genera that were detected in the high-throughput sequencing.

## Results

### Voltage curves of SMFCs

**Fig. 2:**
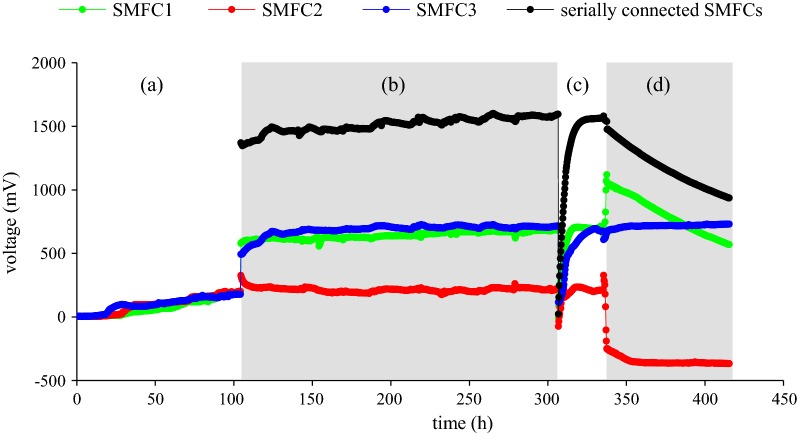
Voltage curve of SMFCs in four stages including **a** acclimation, **b** serially connection, **c** charging a capacitor, and **d** running a timer

The SMFCs were operated with a 1000 Ω external resistor for 110 h to acclimate the exoelectrogenic bacteria until the voltage reached a relatively stable plateau of approximately 185 ± 1 mV, 200 ± 2 mV, and 177 ± 1 mV for SMFC1, SMFC2, and SMFC3, respectively (Fig. 2). Then, the external resistor was removed and the three SMFCs were serially connected. Initially, the OCVs of SMFC1, SMFC2, and SMFC3 were 579 mV, 327 mV, and 493 mV, respectively, and the OCV of the serially connected SMFCs was 1370 mV. During 164 h of serial connection, the OCV of serially connected SMFCs gradually increased to 1595.6 mV. Then, the serially connected SMFCs were used to charge a 1 F capacitor. Immediately after the capacitor was connected to the SMFCs, the OCV sharply decreased, followed by gradual recovery. It took 12 h to fully charge the capacitor, and the voltage of the serially connected SMFCs gradually increased to 1569.1 mV during that time. After the capacitor was charged, an electronic timer was connected in parallel to the capacitor and the serially connected SMFCs. The device kept running for 80 h, until the voltage of the capacitor decreased below 935.8 mV.

### Polarization and power curves

**Fig. 3:**
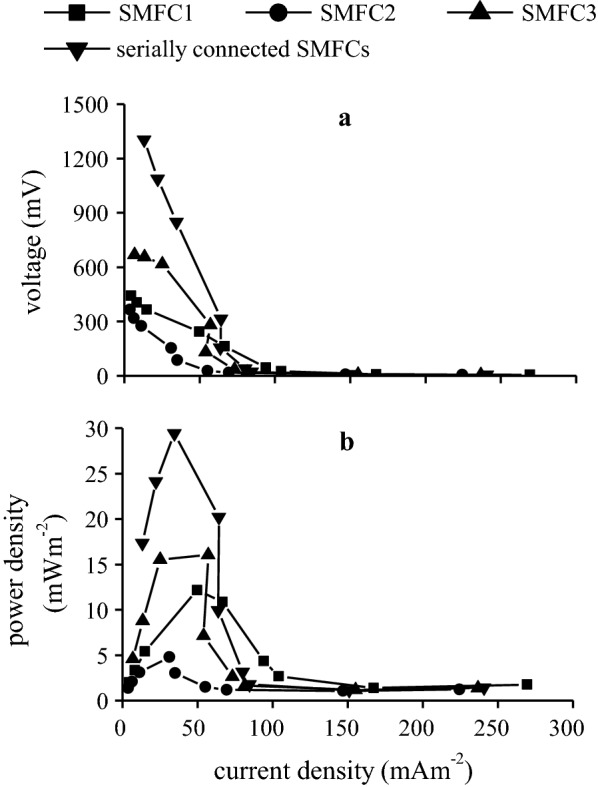
**a** Polarization curves and **b** Power density curves of the individual SMFCs and serially connected SMFCs

The maximum power densities of 12.16, 4.80, and 16.02 mWm^−2^ were achieved at 1000 Ω external resistance for SMFC1, SMFC2, and SMFC3, respectively (Fig. 3). The maximum power density for the serially connected SMFCs was 29.42 mWm^−2^, and it was achieved at an external resistance of 5000 Ω.

### Electrochemical impedance spectroscopy

The Nyquist plots for the two-electrode system and three-electrode system are shown in Additional file 1: Figure S2. An equivalent circuit that comprised two resistor–capacitor parallel circuits and an ohmic resistance was applied to fit the impedance data obtained from the two-electrode system (Additional file 1: Figure S3A). The results demonstrated that the ohmic resistance was 18.4, 21.1, and 12.7 Ω for SMFC1, SMFC2, and SMFC3, respectively (Table 1). However, this mode cannot discriminate anodic *R*_ct_ from cathodic *R*_ct_. Therefore a three-electrode system was applied to determine the cathodic *R*_ct_. An equivalent circuit that comprised one resistor–capacitor parallel circuit and an ohmic resistance was applied to fit the impedance data obtained from the three-electrode system (Additional file 1: Figure S3B). The EIS result of the three-electrode system demonstrated that the cathodic *R*_ct_ was approximately 300–400 Ω, whereas the anodic *R*_ct_ varied from 15.4 to 36.0 Ω for the three SMFCs.

**Table 1:** The internal resistance of the three individual SMFCs

Treatments	*R*_Ω_ (Ω)	Cathodic *R*_ct_ (Ω)	Anodic R_ct_ (Ω)
Two-electrode system			
SMFC1	18.4	428.4	15.4
SMFC2	21.1	323.2	20.9
SMFC3	12.7	325.9	36.0
Three-electrode system			
SMFC1	0.8	443.3	
SMFC2	3.3	310.8	
SMFC3	1.4	353.9	

### Active exoelectrogenic bacteria-associated genera

**Fig. 4:**
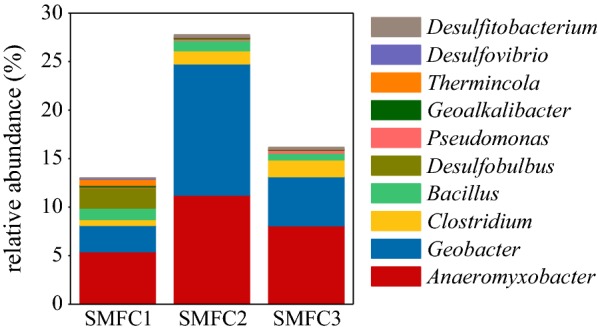
The relative abundance of the active exoelectrogenic bacteria-associated genera detected from the anodes of the three individual SMFCs

Ten active exoelectrogenic bacteria-associated genera were detected from the anode samples of the three SMFCs, including *Anaeromyxobacter*, *Bacillus*, *Clostridium*, *Desulfitobacterium*, *Desulfobulbus*, *Desulfovibrio*, *Geoalkalibacter*, *Geobacter*, *Pseudomonas*, and *Thermincola* (Fig. 4). The genera with a relative abundance > 1% of total bacterial reads in at least one SMFC include *Geobacter* (2.71–13.54%), *Anaeromyxobacter* (5.42–11.23%), *Clostridium* (1.36% in SMFC2 and 1.73% in SMFC3), *Bacillus* (1.19% in SMFC1 and 1.08% in SMFC2) and *Desulfobulbus* (2.12% in SMFC1). The relative abundances of the ten active exoelectrogenic bacteria-associated genera on the anode were 13.03%, 27.78%, and 16.17% for SMFC1, SMFC2, and SMFC3, respectively.

## Discussion

This work demonstrates the use of paddy soil as a power source to successfully run a low-power electric device, following three steps including the acclimation of exoelectrogenic bacteria, serial connection of SMFCs and capacitor charging. Acclimation is necessary to start up the MFCs and it is usually achieved by running the MFCs with a 1000 Ω external resistor (Cheng et al. 2006). In our study, the acclimation process lasted for 110 h, and the voltage of the three SMFCs increased to relatively stable plateaus. After the acclimation process, the three SMFCs were serially connected to obtain a higher voltage than individual SMFCs. We did not observe voltage reversal, which is a problem that sometimes occurs with serial connection (Kim and Chang 2018). This is possibly because straw powder was added into the paddy soil and sufficient substrates prevented the occurrence of voltage reversal (Oh and Logan 2007). Before the capacitor was charged, the serially connected SMFCs reached 1.596 V, which was higher than the 1.5 V generated by one dry battery.

Charging a capacitor is a commonly used method to store electrical power generated from MFCs and to power devices (Zhang et al. 2011; Santoro et al. 2016). When the voltage at the terminals of the capacitor was equal to the voltage of the MFCs, the capacitor was fully charged. In our study, the serially connected SMFCs have an OCV of more than 1.5 V. Therefore, the capacitor could be used to run low-power devices with a rated voltage of no more than 1.5 V. In this regard, such type of devices are very common, including LED lights, sensors, timers, and phone chargers. Our study provides a possibility that these devices could be operated in rice paddy fields without an external battery. However, if the timer was directly connected to the serially connected SMFCs without a capacitor, the SMFCs would fail to run the timer, owing to a voltage collapse (Additional file 1: Figure S4), caused by the high internal resistance of the SMFCs, as explained by the formula:

1 $$U = E - IR_{\text{in}}$$

where *U* is the voltage over a device, *E* is the OCV of the serially connected SMFCs, *I* is the current, and *R*_in_ is the internal resistance. In our study, the internal resistance of each SMFC was approximately 400 Ω, and thus was 1200 Ω for the serially connected SMFCs. This is much higher than the internal resistance of one dry battery.

The high internal resistance also limited the power output of the SMFCs. In our study, the maximum power output of an individual SMFC was 16 mWm^−2^, which is similar to the power output generated by fresh water (e.g. river and lake) sediment-based SMFCs, and approximately half of the power output generated by marine sediment based SMFCs, by using routine materials as anode and cathode (e.g. graphite plate, carbon cloth) (Donovan et al. 2008; Touch et al. 2014; Cai et al. 2016). The applications of a modified anode and cathode (Lowy et al. 2006; Chen et al. 2017, 2018), an amendment with organic substrates (Guo et al. 2018) or a self-stacked submersible configuration (Zhang and Angelidaki 2012) have greatly increased the power density of SMFCs to more than 100 mWm^−2^. Therefore, the performance of the paddy soil-based SMFCs will hopefully be promoted by adopting these methods. In addition, the cost should be considered as well. There have been a number of good alternatives to Pt, such as CoTMPP, Pd, MnO_2_, and carbon nanotubes (Zhao et al. 2005; Gong et al. 2013; Amade et al. 2015). They are much cheaper but have similar performance in catalyzing oxygen reduction, as compared with Pt.

The sequencing result shows that the relative abundance of total active exoelectrogenic bacteria-associated genera on the anode varied between 13.03 and 27.78%. In a previous study, we sequenced 16S rRNA genes of eight different paddy soil samples, including the samples used in the present study. The relative abundance of exoelectrogenic bacteria-associated genera varied between 0.5 and 7.7% in paddy soils (Wang et al. 2019). This indicates that the exoelectrogenic bacteria-associated genera are an active bacterial group on the anode. Moreover, the most active genera on the anode were *Geobacter* and *Anaeromyxobacter*. Together, they accounted for more than 60% of the total active exoelectrogenic bacteria-associated genera. *Geobacter* is a well-known exoelectrogenic bacteria-associated genera (Logan 2009; Chen et al. 2019). A large number of species belonging to *Geobacter* have been identified as exoelectrogenic bacteria, including *G. anodireducens*, *G. bemidjiensis*, *G. bremensis*, *G. chapellei*, *G. humireducens*, *G. hydrogenophilus*, *G. lovleyi*, *G. metallireducens*, *G. sulfurreducens*, and *G. uraniireducens* (Koch and Harnisch 2016). However, our understanding regarding *Anaeromyxobacter* is significantly less than regarding *Geobacter.* Only *A. dehalogenans* has been identified as exoelectrogenic bacteria (Marshall et al. 2009). Therefore, it is hopeful to discover more exoelectrogenic bacteria species belonging to *Anaeromyxobacter*. Moreover, studies in the future could further increase the voltage and power output of SMFCs, and run different electronic devices (such as sensors, lights, and chargers) in the field.

## Additional file

**Additional file 1: Figure S1.** The air temperature in our lab during 420 h (from Sep. 17th to Oct. 5th, 2018) when SMFCs were operated. **Figure S2.** Nyquist plots of the two-electrode system for (A) SMFC1, (B) SMFC2, and (C) SMFC3, and Nyquist plots of the three-electrode system for (D) SMFC1, (E) SMFC2, and (F) SMFC3. **Figure S3.** Equivalent electrical circuits used to fit the EIS data originated from: A) two-electrode system; B) three-electrode system. **Figure S4.** The voltage collapsed after the electronic timer was directly connected to the serially connected SMFCs. The black arrow shows the first voltage data recorded after the direct connection. Voltage data were recorded every 15 min.
