# Supplementary material for: Electricity generation from paddy soil for powering an electronic timer and an analysis of active exoelectrogenic bacteria
Source: AMB Express. 2019 Apr 23;9:57. doi: 10.1186/s13568-019-0781-x (PMC6478775; doi:10.1186/s13568-019-0781-x)
Supplement: Supplementary file 1 — Additional file 1: Figure S1. The air temperature in our lab during 420 h (from Sep. 17th to Oct. 5th, 2018) when SMFCs were operated. Figure S2. Nyquist plots of the two-electrode system for (A) SMFC1, (B) SMFC2, and (C) SMFC3, and Nyquist plots of the three-electrode system for (D) SMFC1, (E) SMFC2, and (F) SMFC3. Figure S3. Equivalent electrical circuits used to fit the EIS data originated from: A) two-electrode system; B) three-electrode system. Figure S4. The voltage collapsed after the electronic timer was directly connected to the serially connected SMFCs. The black arrow shows the first voltage data recorded after the direct connection. Voltage data were recorded every 15 min. [file 13568_2019_781_MOESM1_ESM.docx]

**Additional Material**

**Journal Name**: AMB Express

**Title**: Electricity generation from paddy soil for powering an electronic timer and an analysis of active exoelectrogenic bacteria

Yu Lu^a^, Li Liu^a^, Shaosong Wu^a^, Wenhui Zhong^b,c^, Yujun Xu^d^, Huan Deng^a,b^*

^a^ School of Environment, Nanjing Normal University, Nanjing 210023, China

^b^Jiangsu Provincial Key Laboratory of Materials Cycling and Pollution Control, School of Geography Sciences, Nanjing Normal University, Nanjing 210023, China

^c^ Jiangsu Center for Collaborative Innovation in Geographical Information Resource Development and Application, Nanjing 210023, China

^d^ Honors College, Nanjing Normal University, Nanjing 210023

* **Correspondence**: Huan Deng. School of Environment, Nanjing Normal University, Nanjing 210023, China. Phone: 86 25 85891352; Fax: 86 25 85891347; E-mail address: hdeng@njnu.edu.cn

Number of text pages: 5

Number of tables: 0

Number of figures: 4

Number of color figures: 1 (Fig. S4)

Figure S1 The air temperature in our lab during 420 h (from Sep. 17^th^ to Oct. 5^th^, 2018) when SMFCs were operated.

Figure S2 Nyquist plots of the two-electrode system for (A) SMFC1, (B) SMFC2, and (C) SMFC3, and Nyquist plots of the three-electrode system for (D) SMFC1, (E) SMFC2, and (F) SMFC3.


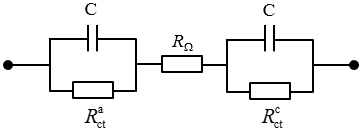


A


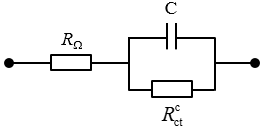


B

Figure S3 Equivalent electrical circuits used to fit the EIS data originated from: A) two-electrode system; B) three-electrode system.

Figure S4 The voltage collapsed after the electronic timer was directly connected to the serially connected SMFCs. The black arrow shows the first voltage data recorded after the direct connection. Voltage data were recorded every 15 min.
